# Independent Prognostic and Predictive Role of Interstitial Macrophages in Kidney Biopsies of IgA Nephropathy Patients

**DOI:** 10.3390/jpm13060935

**Published:** 2023-05-31

**Authors:** Francesca Bianca Aiello, Franco Oreste Ranelletti, Marcella Liberatore, Paolo Felaco, Graziano De Luca, Alessia Lamolinara, Francesco Paolo Schena, Mario Bonomini

**Affiliations:** 1 Department of Medicine and Aging Sciences, University G. D’Annunzio, Chieti-Pescara, 66100 Chieti, Italy; m.bonomini@nephro.unich.it; 2 Formerly Unit of Histology, Università Cattolica del Sacro Cuore, 00168 Rome, Italy; francooreste.ranelletti@fastwebnet.it; 3 Department of Oncology, ASL2, SS Annunziata Hospital, 66100 Chieti, Italy; marcella.liberatore@asl2abruzzo.it; 4 UOC Nephrology and Dialysis PO, 64100 Teramo, Italy; paolo.felaco@aslteramo.it; 5 Graziano De Luca UO Clinical Pathology, Val Vibrata Hospital, 64027 Sant’Omero, Italy; graziano.deluca@aslteramo.it; 6 Department of Neurosciences, Imaging and Clinical Sciences, University G. D’Annunzio, Chieti-Pescara, 66100 Chieti, Italy; alessia.lamolinara@unich.it; 7 Department of Emergency and Organ Transplantation, University of Bari, 70121 Bari, Italy; paolo.schena@uniba.it; 8 Schena Foundation, Valenzano, 70010 Bari, Italy

**Keywords:** IgA nephropathy, macrophages, peritubular capillaries, hypertension, glucocorticoids

## Abstract

A relevant percentage of IgAN patients experience a progressive decline in kidney function. According to the KDIGO guidelines, proteinuria and eGFR are the only validated prognostic markers. The role of interstitial macrophages in kidney biopsies of IgAN patients and the outcome of patients treated with renin–angiotensin system inhibitors (RASBs) alone or combined with glucocorticoids were evaluated. Clinical and laboratory records (age, gender, hypertension, hematuria, proteinuria, eGFR, serum creatinine, and therapy), MEST-C parameters of the Oxford classification, C4d deposition, peritubular capillaries, and glomerular and interstitial macrophages in 47 IgAN patients undergoing kidney biopsy consecutively between 2003 and 2016 were examined. A high number of interstitial macrophages significantly correlated with peritubular capillary rarefaction and impairment of kidney function. Cox’s multivariable regression analysis revealed that a value > 19.5 macrophages/HPF behaved as an independent marker of an unfavorable outcome. Patients exhibiting > 19.5 macrophages/HPF treated at the time of diagnosis with RASBs combined with methylprednisolone had an estimated probability of a favorable outcome higher than patients treated with RASBs alone. Thus, a value > 19.5 macrophages/HPF in IgAN biopsies can predict an unfavorable outcome and endorse a well-timed administration of glucocorticoids. Studies evaluating urine biomarkers associated with peritubular capillary rarefaction in patients with marked macrophage infiltration may help personalized treatment decisions.

## 1. Introduction

IgA nephropathy (IgAN), the most frequent primary glomerulonephritis [1,2], is an autoimmune disease influenced by racial, genetic, immunological, and environmental factors [1,2,3]. The diagnosis of IgAN is based on the presence of a predominant mesangial deposition of IgA in the kidney glomeruli [3]. The accumulation of immune complexes formed by aberrantly glycosylated IgA1 and antiglycan antibodies induces inflammation, mesangial cell proliferation, and extracellular matrix synthesis [1,3]. Occasionally, IgAN may be subclinical [3]. It affects mainly young adults and accounts for approximately 20% of kidney biopsies from children [1,2,3,4]. The majority of IgAN patients exhibit a clinical presentation consisting of micro or macrohematuria with or without proteinuria, and a slowly progressive course. A high percentage of these patients (15–40%), eventually experience a decline in kidney function to end-stage kidney disease (ESKD) with a variable rate of progression [4,5]. Clinical and histological parameters have been evaluated in countless studies; nevertheless, it is still challenging to predict the outcome and devise a personalized therapy. Hypertension, high serum creatinine, and proteinuria, for instance, are known prognostic risk factors, but they may simply indicate the extent of the disease at a particular stage [5]. According to the Kidney Disease Improving Global Outcome (KDIGO) practice guidelines, there is no validated prognostic biomarker for IgAN other than proteinuria and eGFR [6]. Therefore, it would be useful to identify lesions with prognostic value. The Oxford classification, formulated in 2009 and subsequently revised, is largely utilized to assess IgAN histological severity [7,8,9]. It includes five parameters demonstrating prognostic value by univariate analyses: mesangial cellularity (M), endocapillary hypercellularity (E), segmental glomerulosclerosis (S), tubular atrophy (T), and crescents (C) [7,8,9]. High S and T scores at the time of the diagnosis point to an advanced clinical stage [5,10] and a high T score has been associated with a poor outcome in studies performed to validate this classification [11]. Interstitial macrophage infiltration in IgAN kidney biopsies has been associated with poor prognosis and severity of the disease since 2006 [12,13,14,15,16,17,18]; however, this parameter is not included in the Oxford classification. Disease-specific urinary biomarkers can be useful as diagnostic and/or prognostic tools, potentially avoiding second biopsies [19,20,21,22,23,24,25,26,27].

In a previous study, we showed for the first time that the interstitial macrophage number positively correlated with hypertension and S1 and T1 scores [17]. In this paper, we sought to build on previous findings to investigate further the prognostic value of interstitial macrophages. Since glucocorticoids potently inhibit macrophage pro-inflammatory functions [28], we also compared the outcomes of patients administered renin–angiotensin blockers (RASBs) alone or in combination with methylprednisolone six-month treatment at the time of diagnosis.

## 2. Materials and Methods

### 2.1. Patients

Clinical and laboratory records of 47 IgAN patients (35 males and 12 females, median age 34 years, mean age 36.68 ± 12.66 years) undergoing kidney biopsy consecutively between January 2003 and December 2016 were reviewed. Patients included in the study were more than 15 years of age and underwent biopsy before any immune-suppressive treatment. Patients with diabetes mellitus, autoimmune diseases, abnormal hypergammaglobulinemia, and liver diseases were excluded from the study. After biopsy, 16 patients were treated with RASBs, 10 with corticosteroids, and 18 with RASBs and corticosteroids. Three patients with normal kidney function, proteinuria < 1 g/24 h, and only mild mesangial hypercellularity received no treatment. Hematuria, proteinuria (g/24 h), serum creatinine level (mg/dL), blood pressure, and estimated glomerular filtration rate (eGFR) (mL/min per 1.73 m^2^) were recorded at the time of biopsy. Hypertension was defined as systolic blood pressure > 135 mm Hg and/or diastolic blood pressure > 85 mm Hg or the use of anti-hypertensive agents. In the kidney biopsies of patients with hypertension, typical vascular lesions were observed. 

Follow-up time was considered as the interval time between kidney biopsy and the last outpatient visit. Hematuria, proteinuria, serum creatinine levels, and eGFR data at the end of follow-up were available for 30 patients. The evaluation of the impairment of the kidney function, based on the eGFR at the time of biopsy and at the end of follow-up (range: 16–92 months, median: 36 months) was performed using the Kidney Disease Outcome Quality Initiative (KDOQI) guidelines, which defines five stages: I: eGFR ≥ 90 mL/min; II: eGFR = 60–89 mL/min; III: eGFR = 30–59 mL/min; IV: eGFR = 15–29 mL/min; V: eGFR ≤ 15 mL/min [29]. A favorable prognosis was ascribed to patients with a stable stage 1 or with an improved stage at the end of follow-up, whereas patients with an unfavorable outcome showed a stage 2 or lower at the time of the diagnosis and no improvement or a worse stage at the end of follow-up. These patients were treated at the time of diagnosis with RASBs (*n* = 11), methylprednisolone (1.5–1 mg/kg/die) in combination with RASBs (*n* = 11), or methylprednisolone (0.5–1 mg/kg/die) (*n* = 5); methylprednisolone was administered for 6 months. Three patients with normal kidney function, proteinuria < 1 g/24 h, and kidney biopsies with only mild mesangial hypercellularity received no treatment.

### 2.2. Histopathology and Immunohistochemistry

Formalin-fixed paraffin-embedded sections were stained with hematoxylin–eosin, PAS, and Masson’s trichrome. Direct immunofluorescence was performed on fresh frozen tissue with FITC-conjugated polyclonal antibodies to detect IgG, IgM, IgA, C3, and fibrinogen (Dako, Glostrup, Denmark). IgAN biopsies were evaluated according to the revised Oxford Classification [7,8,9]. Each glomerulus was scored for: mesangial hypercellularity (M0–M1, M1 indicated hypercellularity in more than 50% of glomeruli), endocapillary hypercellularity (absent/present: E0–E1), segmental glomerulosclerosis (absent/present: S0–S1), tubular atrophy (T0 ≤ 25%, T1 = 26–50% and T2 ≥ 50% of the cortical area), and the presence of crescents (C0 = no crescents, C1 and C2 = crescents in ≤ 25% and > of 25% of glomeruli, respectively). Interstitial fibrosis of the cortical area was assessed by a semiquantitative score as: absent = up to 10%, mild = 6 to 25%, moderate = 26 to 50%, and severe ≥ 50% [14]. Sections were stained for immunohistochemistry using the bond polymer refine detection method (Leica Biosystem, Wetzlar, Germany) after high pH (pH 9.0) buffered pre-treatment (Leica Biosystem), using the murine monoclonal antibody (MoAb) anti-CD68 (Dako), specific for macrophages. Quantification of glomerular macrophages was performed by counting CD68+ cells in each glomerulus and dividing the sum obtained in each biopsy by the number of scored glomeruli (magnification ×40). Interstitial macrophages in the cortical area were counted in 10 high power fields (HPF) and the mean number per HPF was reported (magnification ×40). 

Staining of endothelial cells with anti-CD31 murine MoAb (Dako) was performed using the Ultravision LP polymer detection method (Thermo Fisher Scientific, Freemont, CA, USA) after antigen retrieval by heating the sections in Tris-EDTA buffer (pH 8.0). To control for non-specific staining, the primary antibodies were replaced by irrelevant matched MoAb or immune serum, as appropriate. The mean number of peritubular capillaries (PTC) in the cortical area was evaluated using a Nikon Eclipse Te 2000-U microscope equipped with the Nikon Nis Vers. D ver 5.21 image analysis software. The mean number of PTC was calculated by dividing the total number of PTC by the number of HPF (magnification ×40). The mean number of HPF counted per biopsy was 36.53 ± 22.10. In 45 cases, tissue sections were available to perform C4d immunostaining. Paraffin sections were stained using a polyclonal anti-C4d antibody (Biomedica, Vienna, Austria) as previously described [30]. Biopsies from patients with membranous glomerulonephritis and minimal change disease were used as positive and negative controls, respectively.

### 2.3. Statistical Analyses 

Results are expressed as means ± SE or medians, as appropriate. Wilcoxon/Kruskal–Wallis and Spearman’s rho correlation tests were used for the statistical analysis of the distributions of interstitial macrophage and peritubular capillary values according to clinical and pathological variables. The standard multiple linear regression model, fitted using the ordinary least squares estimation technique, was utilized to explain variations in the number of interstitial macrophages that can be attributed to variations in the explanatory variables. The backwards stepwise procedure, based on corrected Akaike’s information criterion (AIC), was used to include only the necessary explanatory variables in the prognostic model. Assumptions of the ordinary least squares regression model were verified through residual diagnostics. In particular, exogeneity, normality, homoscedasticity, and independence assumptions were assessed by Student’s *t*, Shapiro–Wilk, Breush–Pagan, and Durbin–Watson tests, respectively. The follow-up median time was 50 months (C.I. 95%: 39–75) and the primary end point went from the time of kidney biopsy to the date of the impairment of the kidney function or to the date of the last available information on the patient’s status. All medians and life tables were computed using the product-limit estimate by Kaplan and Meier and the curves were examined by means of the log-rank test. For the univariable and multivariable analyses, continuous variables were converted to binomial variables based on the initial analysis of the distribution of the variable values, and according to the results of ROC analyses. Univariable and multivariable analyses were performed by Cox’s proportional hazards model and the prognostic accuracy was assessed by Harrell’s concordance index (C-index). The proportional hazards assumption was assessed by visual inspection of log-log survival curves and by linear regressions of scaled Schoenfeld residuals versus time. Collinearity was verified by computing variance inflation factors (VIF) from the covariance matrix of parameter estimates. The backwards stepwise procedure, based on the lowest AIC value, was utilized to reduce the variable numbers in the Cox’s regression model. Event-free survival probabilities, given the covariates and follow-up time, were calculated for the model fitted by the reduced multivariable Cox’s regression. A *p* value < 0.05 was considered statistically significant, evaluating the yield of two-tailed statistical tests. Statistical analyses were performed using the JMP version 13.0 (SAS Institute Inc., Cary, NC, USA) and R Studio software version 3.3.3 (R Development Core Team: A language and environment for statistical computing, Vienna, Austria 2011).

## 3. Results

Kidney biopsies from 47 patients showed predominant IgA immunoreactivity in the mesangial area of glomeruli, as required for the diagnosis of IgAN. Immune-reactivities for IgG, IgM, C3, and fibrinogen were observed in 11, 28, 46, and 22 kidney biopsies, respectively. In 45 cases, C4d immunostaining was performed. Glomeruli, mesangial areas, and peritubular capillaries (PTC) were C4d negative. 

Hematuria was not observed in one patient, macrohematuria was present in two, and microhematuria in forty-four. The distribution of interstitial macrophages and PTC according to clinical and pathological parameters at the time of diagnosis are shown in Table 1. Univariable analyses showed that the number of interstitial macrophages/HPF was significantly higher in biopsies of hypertensive patients than in biopsies of non-hypertensive patients (*p* = 0.0087) (Table 1). A positive correlation with serum creatinine levels (*p* = 0.0003) and a negative correlation with eGFR (*p* = 0.0002) were observed (Table 1).

**Figure 1:**
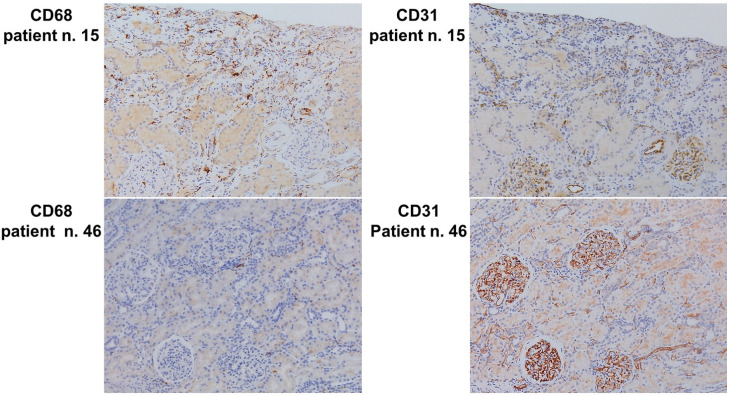
Immunohistochemical staining. Left and right upper panels: CD68-positive macrophages and CD31-positive PTC, respectively (patient n. 15). Numerous macrophages are observed concomitantly with capillary rarefaction (original magnification ×20). Left and right lower panels: CD68-positive macrophages and CD31-positive PTC, respectively, (patient n. 46). Few glomerular CD68+ macrophages are observed concomitantly with normal microvascularization (original magnification ×20).

All biopsies were scored as E0 and C0. The number of interstitial macrophages was not different between M0 and M1 biopsies, whereas it was significantly higher in biopsies scored S1, T1, and with fibrosis than in those scored S0, T0, and without fibrosis (*p* = 0.0102, 0.001, 0.0108, respectively) (Table 1). Fibrosis was scored as severe, moderate, and mild in one, four, and seven patients, respectively. The relationship between PTC and interstitial macrophages has not been previously investigated. We found a significant inverse correlation (*p* = 0.0108) between interstitial macrophages and PTC (Table 1). The PTC of patients with high or low numbers of interstitial macrophages are shown in Figure 1. 

Next, we examined whether the number of PTC/HPF correlated with any other parameter. Interestingly, the mean PTC number/HPF was lower in kidney biopsies of hypertensive patients, with an evident trend toward significance (*p* = 0.054) (Table 1). As in previous studies [13,17] no correlations were observed between the mean number of glomerular macrophages and clinical or pathological parameters. The number of glomerular and interstitial macrophages, however, were positively correlated (*p* = 0.0390) (Table 1). 

**Figure 2:**
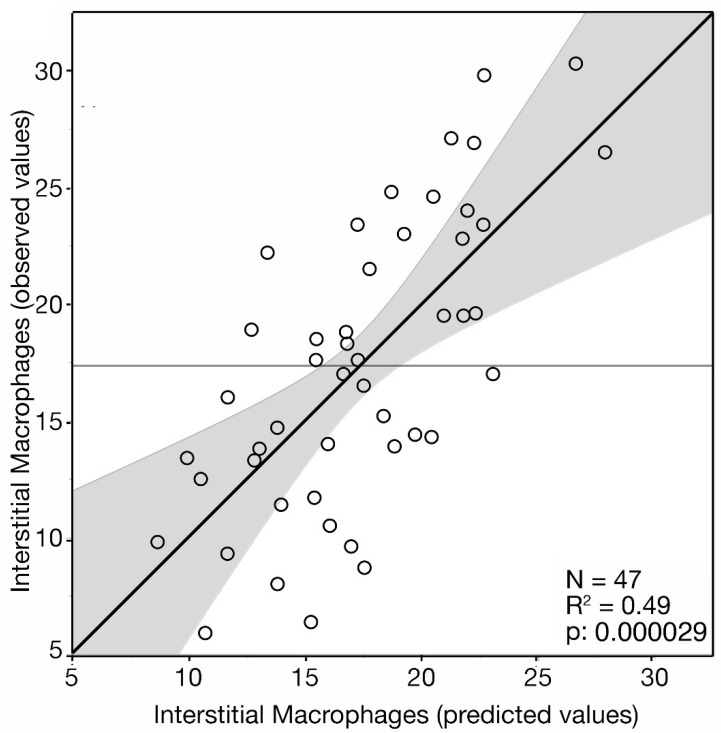
Standard multiple linear regression model. Plot of observed versus predicted interstitial macrophages number/HPF. Leverage plot showing the fit of the actual value of interstitial macrophages/HPF (black line) plotted against their values predicted by the standard multiple linear regression model (open circles). The grey area represents the confidence region at 5% level for the line of the fit. The horizontal line represents the sample mean of the response for the model when the value of the parameters is constrained to zero.

The standard multiple linear regression model was utilized to evaluate the contribution of the clinical and pathological parameters influencing the number of interstitial macrophages. After a backwards stepwise reduction, the variables included in the final predictive model were eGFR, hypertension, an interaction between hypertension and eGFR, segmental glomerulosclerosis, and PTC. The regression of the predicted versus observed interstitial macrophage numbers showed a good predictive performance of the multiple linear regression model (multiple R^2^ = 0.49; F-statistic: 7.871 on 5 and 41 DF, *p*-value: 0.000029), (Figure 2).

**Figure 3:**
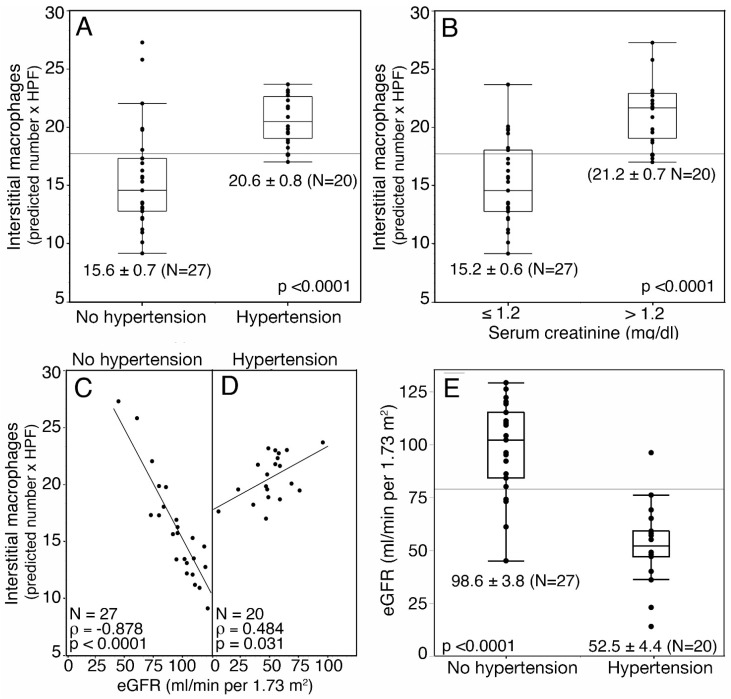

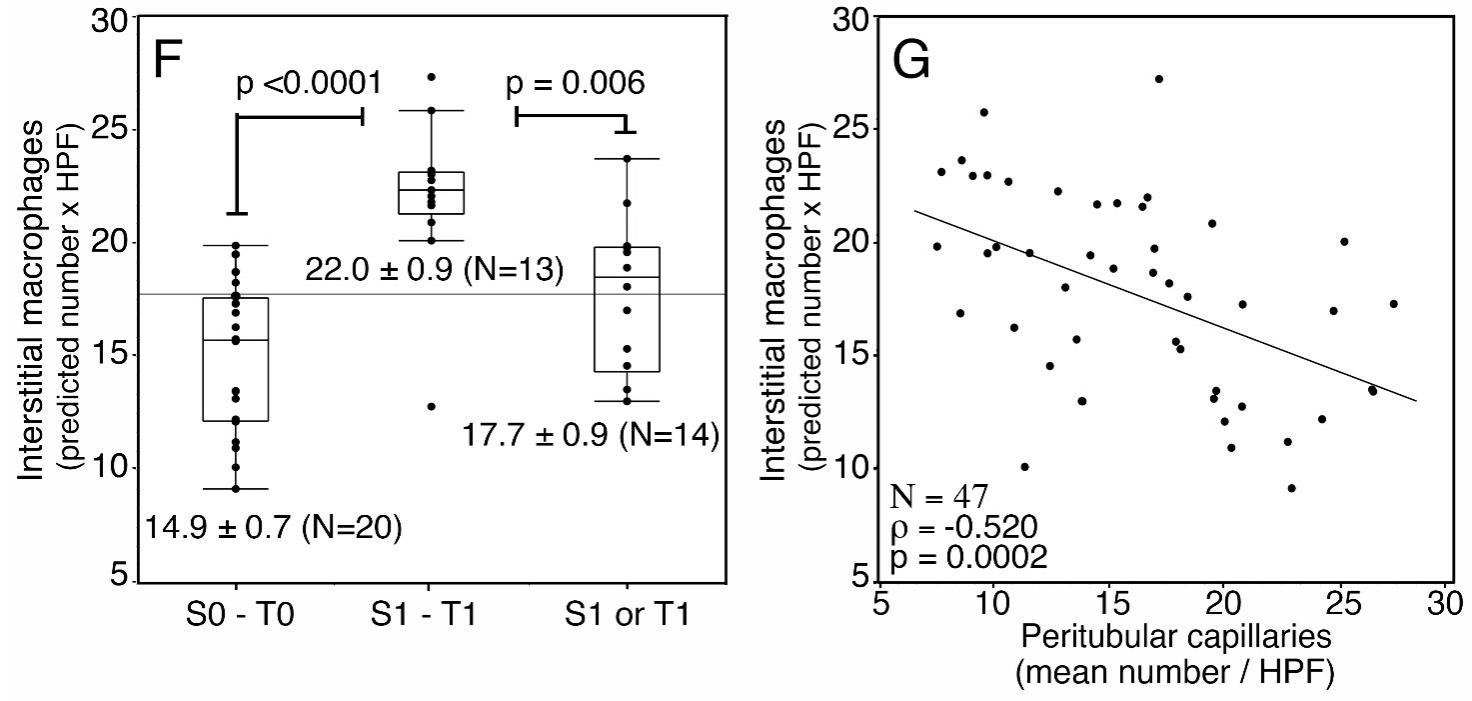
Interstitial macrophages number/HPF predicted from the standard multiple linear regression model according to clinical and pathological stratification of 47 IgAN patients. (**A**) Patients with and without hypertension. (**B**) Patients with serum creatinine ≤ 1.2 mg/dL and > 1.2 mg/dL. (**C**) Plots of the correlations between predicted interstitial macrophages number/HPF and eGFR in patients without hypertension. (**D**) Plots of the correlations between predicted interstitial macrophages number/HPF and eGFR in patients with hypertension. (**E**) Box plot of the eGFR values of patients stratified by the presence or the absence of hypertension. (**F**) Box plot of the correlations between predicted interstitial macrophages number/HPF and S0-T0, S1T1, S0, or T0 scores. (**G**) Plot of correlation between predicted interstitial macrophages number/HPF and PTC. Shown are the regression lines. (**C**,**D**,**G**) Spearman’s rho correlation test.

Based on the regression model, the number of interstitial macrophages, predicted according to the clinical and pathological characteristics of the patients, is shown in Figure 3. Patients with hypertension and serum creatinine values > 1.2 mg/dL showed higher levels of interstitial macrophages (*p* < 0.0001, Figure 3A,B). The interaction between hypertension and eGFR is shown in Figure 3C,D. In patients without hypertension, the predicted number of interstitial macrophages showed a significant inverse correlation with eGFR (*p* < 0.0001) (Figure 3C), whereas, unexpectedly, in hypertensive patients, the correlation between the predicted number of interstitial macrophages and eGFR was positive (*p* < 0.031, Figure 3D). A possible explanation of this result may stem from the observation that the levels of eGFR of patients with hypertension were significantly lower than in patients without hypertension (median: 52 vs. 102; mean: 52.5 vs. 98.6 *p* < 0.0001) (Figure 3E). Then, hypertension, by reducing the eGFR level may interfere with the correlation between interstitial macrophages and eGFR. Patients with both segmental glomerulosclerosis and tubular atrophy, scored S1/T1, had a higher number of predicted interstitial macrophages than patients which scored either S1 or T1 alone (*p* < 0.006) or scored S0/T0 (*p* < 0.0001) (Figure 3F). The number of PTC/HPF was inversely correlated with the predicted number of interstitial macrophages (*p* = 0.0002) (Figure 3G).

**Figure 4:**
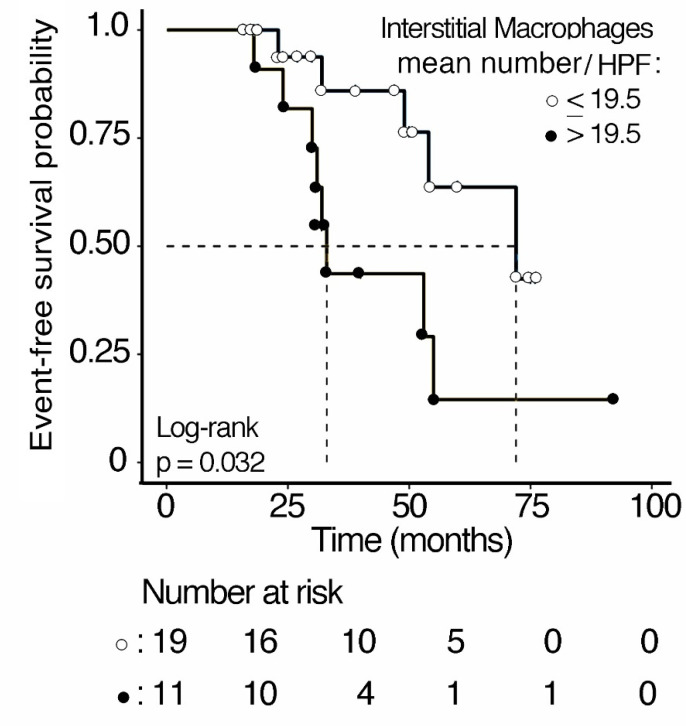
Kaplan–Meier analysis of event-free survival curves of 30 IgAN patients stratified according to interstitial macrophages mean number/HPF in kidney biopsies. The cut-off value was chosen based on an initial analysis of the distribution of the variable values and according to the result of a ROC analysis.

Follow-up data were available for 30 IgAN patients. Thirteen patients (43.3%) experienced an unfavorable outcome (stage 2 or lower at the time of the diagnosis and no improvement or a worse stage at the end of follow-up). The Kaplan–Meier analysis showed that the estimated probability of an unfavorable clinical course was higher for patients with a mean number of interstitial macrophages > 19.5/HPF as compared to patients with a mean number ≤ 19.5/HPF (Figure 4) (*p* = 0.032). At the 5-year follow-up, the estimated proportions of event-free patients were 14.5% ± 12.9 SE and 63.7% ± 15.5 SE for patients with high (>19.5/HPF) and low (≤ 19.5/HPF) mean macrophage number, respectively. Patients with a value > 19.5 macrophages/HPF showed a risk of unfavorable outcomes 3.12 times higher (C.I. 95%: 1.043–1.77) than patients with a value of ≤19.5 macrophages/HPF. 

Cox’s univariable analysis was applied to evaluate the event risk of the prognostic variables. Age ≥ 34 years, serum creatinine > 1.2 mg/dL, eGFR < 76 mL/min per 1.73/m^2^, interstitial macrophage number > 19.5/HPF, and treatment with RASBs alone, were associated with a significantly increased risk of an unfavorable outcome (Table 2). 

**Figure 5:**
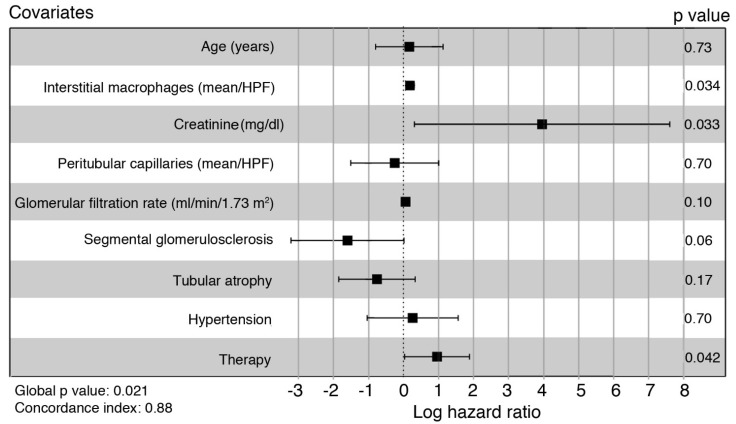
Forest plot of the multivariable Cox’s proportional hazard model. Squares indicate the hazard ratio relative to the reference levels of the covariates, as reported in the univariable analysis. Bars: confidence interval 95%.

Cox’s multivariable regression analysis was utilized to evaluate the relative risk of an unfavorable outcome of the interstitial macrophage number after adjusting for the variables utilized in the univariable analyses. The Cox’s regression model showed a good prognostic accuracy as assessed by Harrell’s concordance index (C-index = 0.88) with a significant global *p*-value (*p* = 0.021). In addition to serum creatinine value > 1.2 mg/dL, and the type of therapy, the mean number of interstitial macrophages/HPF > 19.5/HPF behaved as an independent prognostic marker of an unfavorable outcome (relative risk: 1.16; C.I. 95%: 1.01–1.29; *p* = 0.034) (Figure 5).

**Figure 6:**
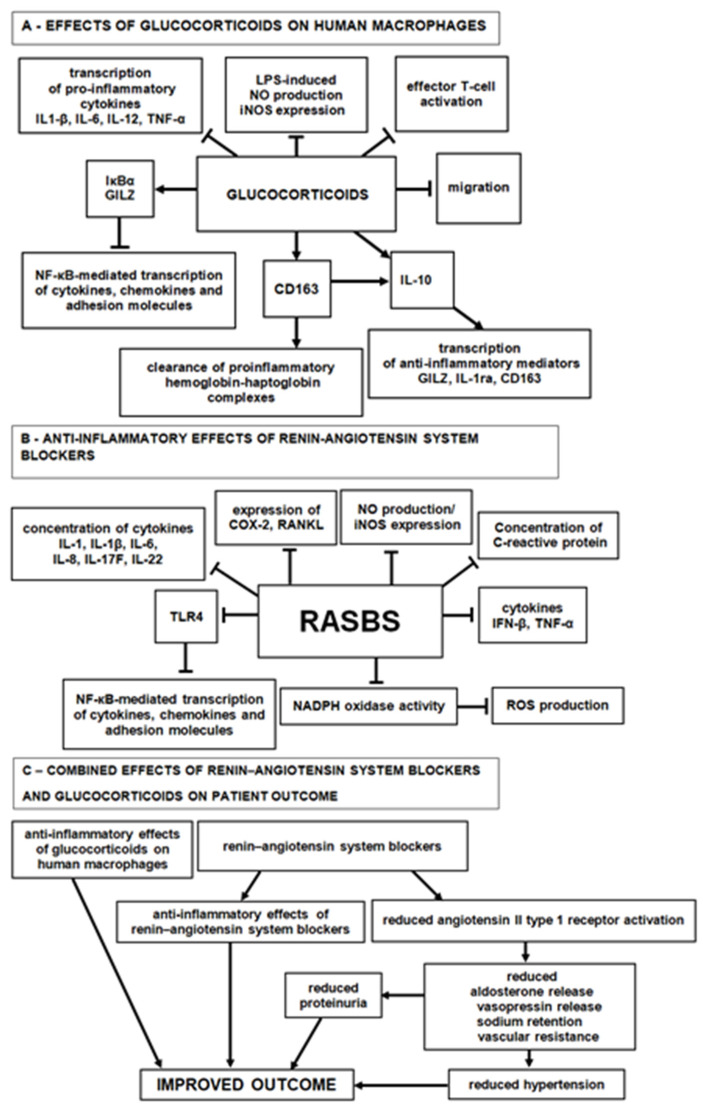
(**A**) Pathways inhibiting pro-inflammatory functions of human macrophages mediated by glucocorticoids; GILZ: glucocorticoid-induced leucine zipper, NO: nitric oxide, iNOS: inducible nitric oxide synthase [28]. (**B**) Reported anti-inflammatory pathways mediated by RASBs on human cells; ROS: reactive oxygen species [34]. (**C**) Hypothetical positive effect on the outcome by converging pathways mediated by a combined treatment with RASBs and glucocorticoids.

Glucocorticoids are well known inhibitors of macrophage pro-inflammatory functions [28], and treatment with prednisolone reduced the number of macrophages in kidney biopsies of IgAN patients [31]. Glucocorticoid anti-inflammatory mechanisms affecting human macrophages are summarized in Figure 6A [28]. 

Inflammation contributes to the development of hypertension [32]. RASBs are effective anti-hypertensive agents [33]. Therefore, they exhibit indirect anti-inflammatory effects. There are reports proposing that they display many additional anti-inflammatory effects on human cells; we have summarized these effects in Figure 6B [34].

As anti-inflammatory pathways underlying a combined therapy could converge (Figure 6C), we compared the outcome of patients treated at the time of diagnosis with RASBs alone or in combination with glucocorticoids.

**Figure 7:**
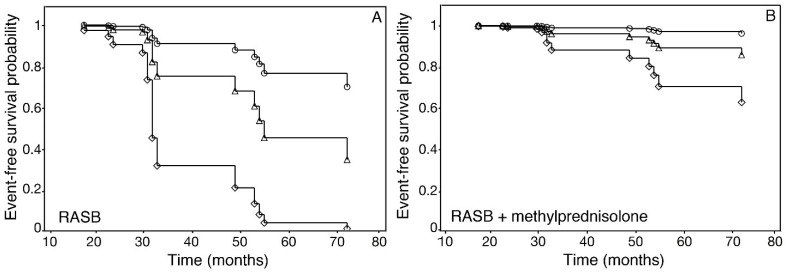
Estimated event-free survival curves for IgAN patients according to interstitial macrophage numbers/HPF and the type of therapy received, after adjusting for baseline prognostic variables in the multivariable Cox’s regression model. (**A**) Patients treated with RASB alone. (**B**) Patients treated with RASB combined with methylprednisolone. Circles = 19 interstitial macrophages/HPF, triangles = 23 interstitial macrophages/HPF, diamonds = 27 interstitial macrophages/HPF, corresponding to Q2, Q3, and Q4 of the interstitial macrophages/HPF value distribution, respectively.

Event-free survival curves based on the Cox regression estimates adjusted at the basal levels of the confounding covariates were calculated as a function of the number of interstitial macrophages corresponding to quartiles Q2 (19/HPF), Q3 (23/HPF), and Q4 (27/HPF), and the type of therapy (RASBs alone or RASBs in combination with methylprednisolone). The estimated event-free survival probabilities were inversely associated with the number of interstitial macrophages in patients treated both with RASBs alone (Figure 7A) and in combination with methylprednisolone (Figure 7B). However, the deleterious effect of the increasing number of macrophages was markedly greater in patients treated with RASBs alone (Figure 7A) as compared with patients treated with RASBs in combination with methylprednisolone (Figure 7B). Thus, at 19, 23, and 27 macrophages/HPF, these patients, compared to those treated with RASBs in combination with methylprednisolone, showed percent reductions of the estimated survival probability of 21%, 49%, and 94%, respectively. 

## 4. Discussion

We report, for the first time, that a marked interstitial macrophage infiltration correlated with microvascular rarefaction in kidney biopsies of IgAN patients. Moreover, interstitial macrophage infiltration was an independent risk factor for a progressive decline in kidney functions. The combination of corticosteroids and RASBs therapy administered after kidney biopsy improved the outcome, particularly when numerous interstitial macrophages were present. 

Our patients did not exhibit active (E, C, C4d deposits) or chronic severe histopathological lesions, and only two patients underwent ESKD at the end of follow-up. Thus, they formed a suitable group to analyze predictive factors of disease progression. Early studies concerning various types of glomerulonephritis have shown that tubulointerstitial damage correlates with progression to ESKD more than glomerular damage [35,36,37]. It is well known that proteinuria stimulates tubular epithelial cells to release the macrophage chemoattractant protein 1 (MCP-1/CCL2) and cytokines activating macrophages in vitro and in vivo [38,39,40,41]. In IgAN, MCP1 is highly expressed by tubular epithelial cells [20,21], and is detected in the urine [23,24,26,27]. Furthermore, its urinary level correlates with tubulointerstitial inflammation, severity of histopathological lesions, and adverse prognosis [23,24,26]. Based on these findings, the damage mediated by macrophages can be an important link between glomerular and interstitial injury.

The reduction in microvascular density or “capillary rarefaction” causes a defective delivery of oxygen/nutrients to the tubules and induces HIF-1-dependent pro-fibrotic mechanisms, including tubular cell–myofibroblast transdifferentiation [42,43,44], thus leading to tubulointerstitial damage [43,44]. Noteworthy, hypoxia stimulates the recruitment of leukocytes and the expression of pro-inflammatory cytokines, creating a vicious circle [38,39]. We have observed that interstitial macrophage infiltration correlated with capillary rarefaction. In addition, an inverse relationship was present between capillary rarefaction and the predicted number of interstitial macrophages. This was observed at the time of diagnosis, suggesting that capillary rarefaction preceded the development of chronic kidney disease, as demonstrated in experimental models of PTC disruption [45,46]. 

In different pathological conditions, distinct mechanisms underlie the microvascular damage [47]. In IgAN, two mechanisms have been investigated: (1) Vascular endothelial growth factor-A (VEGF-A), constitutively expressed in the healthy kidney by podocytes and tubular epithelial cells, is essential to maintain the integrity of PTC [48]. In advanced IgAN, despite capillary rarefaction, this growth factor was markedly expressed by tubular epithelial cells and detected in the urine [49,50]. However, the plasma level of the soluble VEGF-A receptor 1, a negative regulator of VEGF-A activity, was also increased [51]. Importantly, it can be produced by monocytes [52]; (2) The cleavage of collagen XVIII, present in the basement membranes of glomeruli and kidney tubules, produces endostatin, a potent antiangiogenic factor [52]. Endostatin is mainly generated by matrix metalloproteinase-7 [53], released by macrophages in response to a variety of inflammatory mediators [54]. High levels of serum endostatin in IgAN correlated with poor prognosis [27]. Thus, patients with a marked interstitial macrophage infiltrate are likely to exhibit a high amount of urinary antiangiogenic factors. Rarefaction of the microvasculature can precede or follow the development of hypertension [55,56] and can contribute to elevate the vascular resistance in the kidney as in other organs [55]. In addition, there is clear evidence that inflammation precedes and contributes to the development of hypertension [32]. In line with this data, we observed a lower number of PTC in hypertensive patients. Noteworthy, in animal experimental models, RASBs can antagonize PTC rarefaction independently of their anti-hypertensive effect [57]. 

By a multivariable analysis, we have shown, confirming our previous results, that a low eGFR predicted a high macrophage number [18]. In this study, we have observed that this inverse correlation was not present in patients with hypertension. When the relationship between hypertension and eGFR was analyzed, we found that all hypertensive patients exhibited a low eGFR. This is in line with the results of an extensive meta-analysis showing that hypertension is an independent predictor of decreased eGFR [58]. 

A high number of glomerular macrophages does not correlate with clinical or pathological parameters [14,17]. However, it has been reported to predict a positive response to immunosuppressive therapies, and it may indicate acute inflammation [59]. Interestingly, in this study, we have observed a positive correlation between the number of glomerular and interstitial macrophages.

To date, there are no univocal indications for treating IgAN patients with glucocorticoids.

It has been proposed that distinct MEST-C scores could be exploited to suggest a personalized immunosuppression [60,61,62,63,64]. These include E1 [61], M1 [60], particularly in children [63], S1, particularly if associated with podocyte hypertrophy [64], and C1–2, particularly in association with E1 [65]. In our study, the concurrency of S1 with T1 predicted more interstitial macrophages than each single score. 

According to the KDIGO guidelines, however, the evidence to support an immunosuppressive treatment decision based on the MEST-C criteria is insufficient, and only patients showing proteinuria > 1 g/die following a six-month treatment with RASBs are at high risk of progression and should be considered for the immunosuppressive therapy [6]. However, the extent of proteinuria may be influenced by hemodynamic factors that are not always determined by immunological injuries and would not be affected by an immunosuppressive treatment [66,67]. Thus, proteinuria may not be the only useful criterion to stratify patients for a personalized therapeutical approach [67]. Three of the four clinical trials performed so far have shown the effectiveness of glucocorticoids [68]. A well-timed administration of RASBs combined with glucocorticoids may generate converging pathways to reduce proteinuria, hypertension, and macrophage-mediated inflammation.

At the time of diagnosis, we administered a six-month treatment with glucocorticoids alone when a marked interstitial inflammatory infiltrate was observed in the absence of hypertension. RASBs alone were administered in the presence of proteinuria > 1 g/die and/or hypertension without a marked interstitial inflammation. RASBs in combination with a six-month treatment with methylprednisolone were administered when proteinuria was >1 g/die and/or hypertension were associated with active lesions and/or marked interstitial inflammation. The combined therapy clearly reduced the deleterious effect of increasing amounts of interstitial macrophages, suggesting that a timely addition of glucocorticoids in selected patients was advantageous. 

A limitation of this study is the relatively small number of patients with follow-up data. In addition, being a retrospective analysis, it was not possible to investigate the mechanisms underlying PTC rarefaction. Nonetheless, we have shown that a high number of interstitial macrophages in IgAN is an independent prognostic indicator for the risk of an unfavorable outcome and could endorse the choice of a well-timed administration of glucocorticoids.

Thus, the number of CD68-positive cells should be routinely evaluated, and prospective studies concerning urinary biomarkers connected with this lesion are desirable to support personalized treatment decisions at the time of biopsy, and throughout the follow-up.

## Figures and Tables

**Table 1:** Distribution of interstitial macrophages and peritubular capillaries according to clinical and pathological parameters.

Interstitial Macrophages					Peritubular Capillaries	
Variables		*n*	Median (IQR) ^1^	Wilcoxon *p*	Median (IQR) ^1^	Wilcoxon *p*
hypertension	absent	27	14.7 (10.1–20.6)	0.0087	17.9 (13.1–20.8)	0.054
	present	20	19.6 (17.1–23.7)		14.4 (9.8–17.5)	
proteinuria	<0.5 g/die	5	9.8 (9.2–13.9)	0.014	16.9 (13.4–21.7)	0.52
	>0.5 g/die	42	18.0 (13.9–23.1)		15.9 (10.8–19.8)	
mesangial hypercellularity	M0	36	16.8 (11.5–22.7)	0.22	17.1 (10.7–20.3)	0.35
	M1	11	18.3 (14.7–23.4)		13.6 (11.6–15.4)	
segmental glomerulosclerosis	S0	26	15.6 (10.3–18.6)	0.0102	17.8 (11.5–21.3)	0.17
	S1	21	19.6 (14.1–25.6)		14.6 (10.2–17.7)	
tubular atrophy	T0	28	14.2 (10.7–17.6)	<0.0001	17.8 (13.7–20.7)	0.067
	T1	19	22.2 (18.5–26.5)		13.1 (9.8–17.2)	
fibrosis	absent	35	15.2 (11.7–19.6)	0.0108	17.0 (11.6–20.8)	0.18
	present	12	21.75 (17.4–26.1)		14.6 (10.3–17.0)	
**Interstitial macrophages**					**Peritubular capillaries**	
Variables		*n*	Spearman r	*p*	Spearman r	*p*
creatinine mg/dL		47	0.5008	0.0003	0.2199665	0.14
eGFR (mL/min per 1.73/m^2^)		47	−0.5200	0.0002	−0.2756926	0.061
glomerular macrophages (mean/glomerulus)		47	0.3022	0.0390	−0.2196074	0.14
interstitial macrophages (mean/HPF)		47			−0.3685	0.0108

^1^ IQR = interquartile range.

**Table 2:** Cox’s univariable analysis of prognostic variables in 30 IgAN patients.

Variables		*n*	RR ^1^	CI ^2^	*p ^3^*
Age	<34	15	1.0	(1.5–36.3)	0.008
	>34	15	5.6		
Gender	female	6	1.0	(0.3–4.7)	0.95
	male	24	1.1		
Hypertension	absent	17	1	(0.5–4.7)	0.56
	present	13	1.4		
Creatinine (mg/dL)	≤1.2	17	1.0	(1.0–15.5)	0.042
	>1.2	13	3.4		
eGFR (ml/min per 1.73/m^2^)	>76	14	1.0	(1.3–31.7)	0.016
	≤76	16	4.3		
Proteinuria g/die	≤0.5	4	1	(0.3–30.5)	0.6
	>0.5	26	1.7		
Segmental glomerulosclerosis	S0	15	1	(0.4–5.2)	0.56
	S1	15	1.4		
Tubular atrophy	T0	19	1	(0.7–7.3)	0.17
	T1	11	2.19		
Fibrosis	absent	23	1.0	(0.2–2.6)	0.75
	present	7	0.8		
Interstitial macrophages/HPF	≤19.5	19	1.0	(1.1–10.7)	0.038
	>19.5	11	3.2		
Peritubular Capillaries/HPF	>17.2	11	1.0	(0.6–62.8)	0.18
	≤17.2	19	3.3		
Therapy	RASBs + steroids	11	31	(1.2–17.3)	0.028
	RASBs	16	3.83		

^1^ Unadjusted relative risk; ^2^ 95% confidence intervals; ^3^ Likelihood Ratio Tests.

## Data Availability

Derived data supporting the findings of this study are available from the corresponding author on request.
